# The first global deep-sea stable isotope assessment reveals the unique trophic ecology of Vampire Squid *Vampyroteuthis infernalis* (Cephalopoda)

**DOI:** 10.1038/s41598-019-55719-1

**Published:** 2019-12-13

**Authors:** Alexey V. Golikov, Filipe R. Ceia, Rushan M. Sabirov, Jonathan D. Ablett, Ian G. Gleadall, Gudmundur Gudmundsson, Hendrik J. Hoving, Heather Judkins, Jónbjörn Pálsson, Amanda L. Reid, Rigoberto Rosas-Luis, Elizabeth K. Shea, Richard Schwarz, José C. Xavier

**Affiliations:** 1 Department of Zoology, Kazan Federal University, 420008 Kazan, Russia; 2 Marine and Environmental Sciences Centre, Department of Life Sciences, University of Coimbra, 3000-456 Coimbra, Portugal; 3 Department of Life Sciences, Natural History Museum, SW7 5BD London, UK; 4 Graduate School of Agricultural Science, Tohoku University, 980-0845 Sendai, Japan; 5 Collections and Systematics Department, Icelandic Institute of Natural History, 210 Gardabaer, Iceland; 6 GEOMAR, Helmholtz Centre for Ocean Research Kiel, 24105 Kiel, Germany; 7 Department of Biological Sciences, University of South Florida St. Petersburg, 33701 St. Petersburg, FL USA; 8 Marine and Freshwater Research Institute, 101 Reykjavik, Iceland; 9 Australian Museum Research Institute, 2010 Sydney, NSW Australia; 10 CONACyT-Tecnológico Nacional de México/I.T.Chetumal, 77013 Chetumal, Quintana Roo México; 11 Tecnologico Nacional de Mexico/I. T. Chetumal, 77013 Chetumal, México; 12 Delaware Museum of Natural History, 19807 Wilmington, DE USA; 13 Escola do Mar, Ciência e Tecnologia, Universidade do Vale do Itajaí, 88302901 Itajaí, Brazil; 14 British Antarctic Survey, Natural Environment Research Council, CB3 0ET Cambridge, UK

**Keywords:** Zoology, Biooceanography, Stable isotope analysis

## Abstract

*Vampyroteuthis infernalis* Chun, 1903, is a widely distributed deepwater cephalopod with unique morphology and phylogenetic position. We assessed its habitat and trophic ecology on a global scale via stable isotope analyses of a unique collection of beaks from 104 specimens from the Atlantic, Pacific and Indian Oceans. Cephalopods typically are active predators occupying a high trophic level (TL) and exhibit an ontogenetic increase in *δ*^15^N and TL. Our results, presenting the first global comparison for a deep-sea invertebrate, demonstrate that *V. infernalis* has an ontogenetic decrease in *δ*^15^N and TL, coupled with niche broadening. Juveniles are mobile zooplanktivores, while larger *Vampyroteuthis* are slow-swimming opportunistic consumers and ingest particulate organic matter. *Vampyroteuthis infernalis* occupies the same TL (3.0–4.3) over its global range and has a unique niche in deep-sea ecosystems. These traits have enabled the success and abundance of this relict species inhabiting the largest ecological realm on the planet.

## Introduction

Assessing the structure of complex food webs in the world’s oceans is crucial to our integrative understanding of marine ecosystems^1,2^. The trophic interactions among fauna inhabiting the meso- and bathypelagic zones, the two largest and least explored biotopes of the world, are particularly poorly understood^3,4^. Cephalopods are one of the most abundant and widespread animal groups in deep-sea ecosystems, and more than 80% of cephalopod families include deepwater species or live entirely in the deep sea^5^. Cephalopods are important prey and predators in deep-sea ecosystems^6^, and some deepwater squid families (e.g. Mastigoteuthidae and Cranchiidae) are especially high in total biomass^6,7^.

Despite its common name, the Vampire Squid, *Vampyroteuthis infernalis* Chun, 1903 (Cephalopoda, Vampyromorpha) is not a squid, but a unique animal with some traits common to those found in squids and others similar to those of octopods^8–11^. Phylogenetic studies have placed *V. infernalis*, together with the Octopoda, in the clade Octopodiformes^12–16^, concluding that it diverged from the octopods soon after the separation of Octopodiformes and Decapodiformes in the early Triassic^17^. It is small compared to some other oceanic cephalopods, with a maximum reported mantle length (ML) of 210 mm^18^, and is gelatinous in consistency.

*Vampyroteuthis infernalis* is the only extant species of Family Vampyroteuthidae, and has a worldwide distribution in temperate and tropical regions of the Atlantic, Pacific and Indian Oceans^19–21^. It has been recorded up to 57.1°N on the Mid-Atlantic Ridge^7^ and up to 51.3°N in the Pacific^22^. The vertical distribution of *V. infernalis* covers meso- and bathypelagic zones from 600 to 3300 m depth, where it is commonly, but not exclusively, associated with oxygen minimum zones (OMZ)^18–20,23–25^ (Judkins *et al*. unpublished data). Adaptations for living in the OMZ relate to minimizing energy expenditure and include: suppression of aerobic metabolism resulting in the lowest mass-specific metabolic rate among cephalopods^26^; the use of a haemocyanin (respiratory protein) with the highest affinity for O_2_ among all cephalopod heamocyanins investigated so far^24^, except for one benthic octopus from the Antarctic^27^; neutral buoyancy to reduce the energy costs of swimming^26,28^; and using retractile filaments to collect detritus and small planktonic organisms, along with cirri on the arms to manipulate captured food towards the mouth^18^.

Cephalopods are typically active predators^6,29^ and *V. infernalis* was reported to be planktophagous^8^. However, other more recent studies have found evidence for an opportunistic detritivorous feeding mode (i.e. feeding on ‘marine snow’, particulate organic matter (POM)^18^). Analysis of stomach contents has revealed that, apart from detritus (e.g. fecal pellets of zooplankton, larvacean houses, radiolarians and diatoms), the remains of large prey are often found: pieces of gelatinous plankton, whole and pieces of mesozooplanktonic crustaceans, fish scales and bones^8,18^ (Seibel, pers. comm.) (Supplementary Table 2). While *V. infernalis* has usually been observed passively floating and collecting detritus and small planktonic organisms with the arm filaments^18^, there are also occasional observations and indirect suggestions based on stomach contents analysis corroborating the consumption of (pieces of) larger fast moving prey: crustaceans, squids and even fishes^8,18^ (Seibel, pers. comm.) (Supplementary Table 2). However, most of these studies are based on limited data from Northern Hemisphere observations; its trophic ecology over its global range is not clear, and additional information obtained by other methods is required.

Stable isotope analysis (SIA) is a frequently used method in trophic ecology studies^30–32^, where *δ*^13^C typically provides information on foraging habitat and *δ*^15^N on the trophic level (TL) occupied (e.g.^30,33^). Despite a steady increase in the number of SIA studies performed on a variety of marine taxa and communities, these studies are mostly regional (reviews^31,32^:). To our knowledge there are only three global-scale SIA studies of marine species^34–36^, and none is focused on deepwater species.

The few regional SIA studies that include *V. infernalis* have found contradictory evidence regarding its habitat and trophic ecology (e.g. *δ*^13^C ranging from –21.0‰ to –17.0‰ and *δ*^15^N from 6.7‰ to 14.0‰ in specimens collected from different oceans^37–39^;Seibel *et al*., unpublished data). Using data from the Bay of Biscay^37^, the TL for *V. infernalis* was estimated to be around 4.6–4.7^40^, which is surprisingly high for a POM-eating organism. An ontogenetic increase in *δ*^13^C, *δ*^15^N and TL is typical for cephalopods studied to date^33,41–45^, corresponding to the consumption of prey of higher TLs over their lifespans (review^6^). However, we propose the hypothesis that this ontogenetic increase in *δ*^13^C, *δ*^15^N and TL does not apply to *V. infernalis* due to their unique trophic ecology.

**Figure 1:**
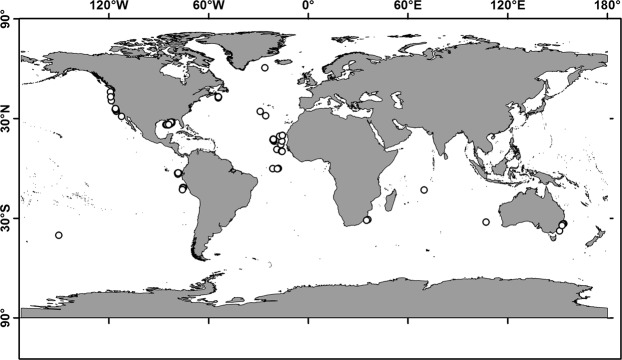
*Vampyroteuthis infernalis* sample locations. Map created by A.V.G. in QGIS 3.8.0 (QGIS Development Team, 2009. QGIS Geographic Information System. Open Source Geospatial Foundation. http://qgis.org).

In the present study, SIA is applied to 104 specimens of *V. infernalis* from the Atlantic, Pacific and Indian Oceans (Fig. 1), including four ontogenetic groups (paralarvae, small, medium and large specimens; Table 1, Supplementary Table 1). The aim is to assess their foraging habitat and TLs throughout their worldwide range. This study is the first to investigate global isotopic patterns for a deep-sea invertebrate.

**Table 1:** Mantle length (ML), values of *δ*^13^C and *δ*^15^N and estimated trophic level (TL) and its coefficient of variation (CV) in *Vampyroteuthis infernalis*. Values are minimum – maximum (mean ± standard error).

Stage	All specimens						Atlantic Ocean					
	n	ML, mm	*δ*^13^C, ‰	*δ*^15^N, ‰	TL	CV	n	ML, mm	*δ*^13^C, ‰	*δ*^15^N, ‰	TL	CV
All	104	6–198 (68.7 ± 4.5)	−20.7– −15.8 (−17.8 ± 0.1)	5.9–16.1 (9.4 ± 0.2)	3.0–4.3 (3.7 ± 0.04)	10%	66	6–133 (45.5 ± 4.0)	−20.7– −16.3 (−18.2 ± 0.1)	5.9–11.8 (8.3 ± 0.2)	3.0–4.3 (3.8 ± 0.04)	8%
Paralarvae	27	6–26 (17.7 ± 1.2)	−20.7– −16.5 (−18.2 ± 0.2)	7.1–11.8 (9.1 ± 0.3)	3.3–4.3 (3.9 ± 0.04)	6%	27	6–26 (17.7 ± 1.2)	−20.7– −16.5 (−18.2 ± 0.2)	7.1–11.8 (9.1 ± 0.3)	3.3–4.3 (3.9 ± 0.04)	6%
Small	24	27–63 (42.1 ± 2.3)	−19.6– −16.5 (−18.2 ± 0.1)	5.9–14.6 (8.7 ± 0.4)	3.0–4.3 (3.8 ± 0.1)	8%	19	27–63 (39.6 ± 2.1)	−19.6– −17.0 (−18.3 ± 0.1)	5.9–9.7 (8.0 ± 0.3)	3.0–4.3 (3.8 ± 0.1)	7%
Medium	26	66–104 (80.8 ± 20.1)	−19.1– −15.9 (−17.9 ± 0.2)	5.9–13.5 (8.7 ± 0.5)	3.0–4.3 (3.6 ± 0.1)	9%	16	66–104 (80.6 ± 2.6)	−19.1– −17.4 (−18.4 ± 0.1)	5.9–8.6 (7.3 ± 0.2)	3.3–4.0 (3.6 ± 0.1)	5%
Large	27	105–198 (131.8 ± 3.9)	−19.6– −15.8 (−17.0 ± 0.2)	7.1–16.1 (11.0 ± 0.5)	3.0–4.3 (3.7 ± 0.1)	13%	4	105–133 (121.8 ± 6.2)	−17.5– −16.3 (−16.9 ± 0.3)	7.8–9.3 (8.7 ± 0.3)	3.2–4.2 (3.7 ± 0.2)	12%
**Stage**	**Pacific Ocean**						**Indian Ocean**					
	**n**	**ML, mm**	***δ***^**13**^**C, ‰**	***δ***^**15**^**N, ‰**	**TL**	**CV**	**n**	**ML, mm**	***δ***^**13**^**C, ‰**	***δ***^**15**^**N, ‰**	**TL**	CV
All	34	27–198 (110.1 ± 6.2)	−19.6– −15.8 (−17.1 ± 0.2)	7.1–16.1 (11.5 ± 0.5)	3.0–4.3 (3.7 ± 0.1)	13%	4	59–145 (100.1 ± 23.1)	−18.2– −16.5 (−17.5 ± 0.4)	8.8–11.2 (9.8 ± 0.5)	3.4–4.0 (3.8 ± 0.1)	7%
Paralarvae	0	—	—	—	—	—	0	—	—	—	—	—
Small	3	27–61 (45.9 ± 10.0)	−18.5– −17.9 (−18.2 ± 0.2)	9.1–14.6 (12.2 ± 1.6)	3.2–4.2 (3.9 ± 0.3)	14%	2	59–62 (60.3 ± 1.2)	−18.2– −16.5 (−17.3 ± 0.9)	10.0–11.2 (10.6 ± 0.6)	3.4–3.8 (3.6 ± 0.3)	8%
Medium	10	66–101 (81.3 ± 3.8)	−18.7– −15.9 (−17.1 ± 0.3)	7.4–13.5 (11.0 ± 0.8)	3.0–4.3 (3.7 ± 0.2)	13%	0	—	—	—	—	—
Large	21	110–198 (133.0 ± 3.8)	−19.6– −15.8 (−17.0 ± 0.2)	7.1–16.1 (11.7 ± 0.6)	3.0–4.3 (3.6 ± 0.1)	14%	2	134–145 (139.9 ± 5.5)	−17.9– −17.4 (−17.6 ± 0.2)	8.8–9.2 (9.0 ± 0.2)	3.9–4.0 (3.95 ± 0.05)	2%

## Results

Our specimen from the Mid-Atlantic Ridge (61.5°N, 30.4°W; *n* = 1; Supplementary Table 1) represents the current northernmost record of the species. It was collected almost 500 km north of the previously known northernmost location^7^.

### Values of *δ*^13^C of *Vampyroteuthis infernalis*

**Figure 2:**
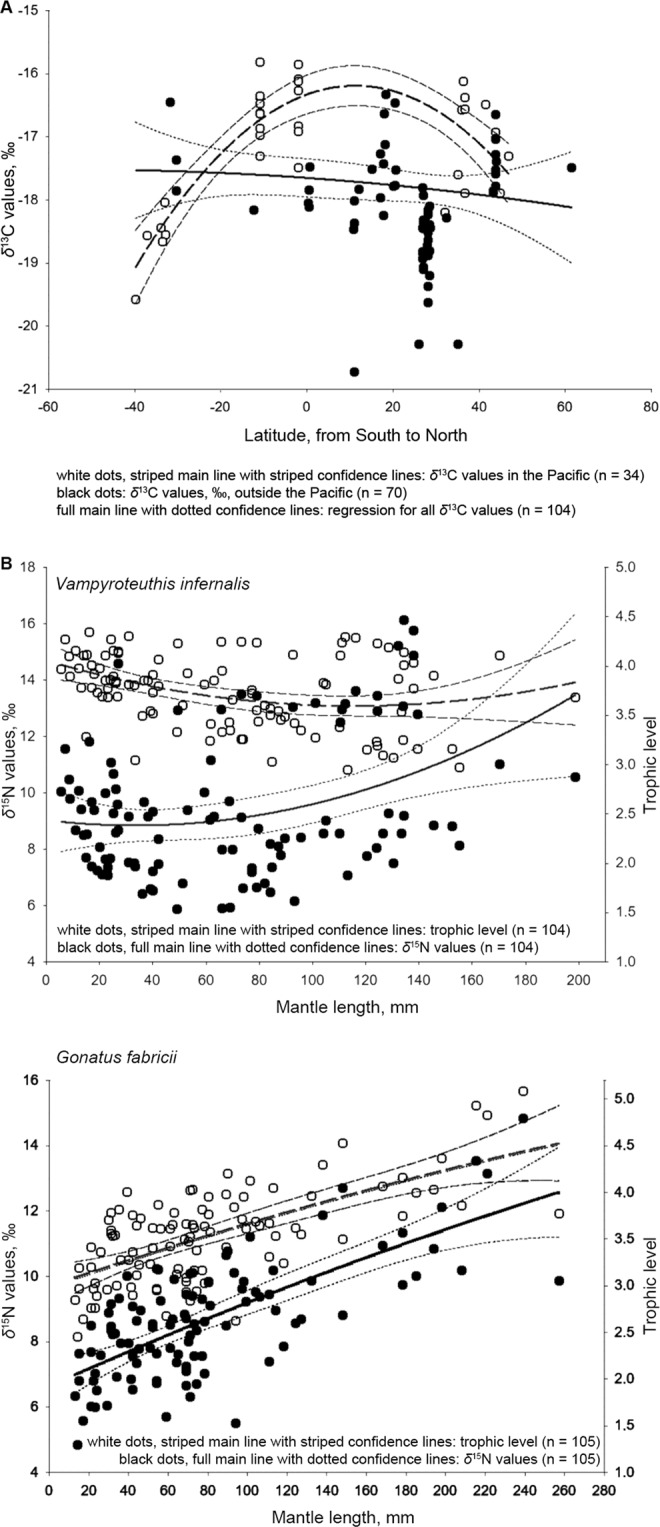
Ontogenetic changes in stable isotope values and trophic levels in *Vampyroteuthis infernalis*. (**A**) Values of *δ*^13^C. (**B**) Values of *δ*^15^N and trophic levels. Data on *Gonatus fabricii* are shown as an example of ontogenetic increase in typical carnivorous squids (modified from^44^). Confidence intervals are 95%.

The overall range of *δ*^13^C was 4.9‰ (i.e. mean ± SE was –17.8 ± 0.1‰; Fig. 2A, Table 1), with significant differences found among the three oceans (*H*_2,104_ = 25.80, *d* = 1.11, *p* < 0.001). Values were significantly higher in the Pacific (–17.1 ± 0.2‰) than in the Atlantic (–18.2 ± 0.1‰; *U* = 441.5, *d* = 1.13, *p* < 0.001). For the three oceans overall, the relationship of *δ*^13^C with habitat depth revealed a weak but not significant decrease in *δ*^13^C values with increasing depth, from 250 m to 1750 m (Spearman *r* = –0.26, *p* = 0.057). Considering each ocean separately, no relationships with depth were found (Spearman *r* = –0.22, *p* = 0.12 for the Atlantic; Pearson *r* = –0.12, *p* = 0.78 for the Pacific; too few specimens from the Indian Ocean were available to obtain reliable results).

No significant linear relationships of *δ*^13^C with latitude were found overall, and the range of *δ*^13^C was 3.8‰ in the Atlantic and 4.4‰ in the Pacific (Fig. 2A, Table 1). Data from the Pacific showed an expected significant latitudinal pattern (*p* = 0.001, if linear) with increasing values towards the equator and decreasing values towards the poles (*r*^2^ = 0.65, intercept –16.38, slopes –0.001 and 0.03; Fig. 2A). However, in the Atlantic (all data collected in the Northern Hemisphere), no significant linear relationship was found.

A significant ontogenetic increase in *δ*^13^C was detected (Pearson *r* = 0.48, *p* < 0.001). Significant differences in *δ*^13^C values were found among ontogenetic groups (*F*_3,100_ = 10.62, *d* = 0.49, *p* < 0.001), with large specimens showing significantly higher values than paralarvae (*Q* = 7.07, *d* = 1.60, *p* < 0.001), small (*Q* = 6.61, *d* = 1.59, *p* < 0.001) or medium (*Q* = 4.89, *d* = 1.00, *p* = 0.004) specimens (Table 1). There was no significant linear ontogenetic relationship in the Atlantic (Pearson *r* = 0.19; *p* = 0.12), with a significant pattern of ontogenetic differences, repeating the global pattern shown above (*F*_3,62_ = 3.54, *d* = 1.68, *p* = 0.020). Both linear trend (Pearson *r* = 0.32, *p* = 0.06) and overall pattern (*F*_2,31_ = 2.41, *d* = 1.33, *p* = 0.11) of ontogenetic differences were not significant in the Pacific Ocean (Table 1). Comparing ontogenetic groups between the Atlantic and Pacific, significantly higher *δ*^13^C values were found in the Pacific in medium specimens (*U* = 20, *d* = 1.58, *p* = 0.001), while values in small and large specimens were very similar, but paralarvae were sampled only in the Atlantic (Table 1). Four available specimens from the Indian Ocean were included for general comparison only (Table 1).

In relation to OMZs (definitions of oxygenation conditions applied in this paper are detailed in Materials), the lowest values of *δ*^13^C were detected in specimens inhabiting ‘normal’ oxygenation conditions (–18.3 ± 0.1‰), followed by oxygen limited zones (OLZs; –17.8 ± 0.1‰) and the highest in OMZs (–17.1 ± 0.1‰), with significant differences between specimens from ‘normal’ and OMZs (*H*_2,104_ = 42.15, *d* = 1.63, *p* < 0.001; *U* = 302, *d* = 1.65, *p* < 0.001) (Table 2, Supplementary Table 3). Both in the Atlantic and in the Pacific, specimens from OMZs showed significantly higher *δ*^13^C values than in ‘normal’ conditions (*U* = 168.5, *d* = 0.80, *p* = 0.003 and *U* = 1, *d* = 1.68, *p* < 0.001, respectively). Also, the values from OMZs were higher than in ‘normal’ conditions in all ontogenetic groups, overall and from the Atlantic and the Pacific (Table 2, Supplementary Table 3).

**Table 2:** Differences in mean values of *δ*^13^C and *δ*^15^N and estimated trophic level (TL) in *Vampyroteuthis infernalis* from different oxygenation conditions and from different areas: oxygen minimum zones (OMZ) and ‘normal’ (Normal) (see Methods for O_2_ concentrations).

Stages	Conditions	n	*δ*^13^C, ‰	*δ*^15^N, ‰	TL
All specimens					
All stages	OMZ	43	−17.1 ± 0.1	11.2 ± 0.4	3.8 ± 0.1
	Normal	55	−18.4 ± 0.1	8.1 ± 0.2	3.7 ± 0.04
	Differences		*U* = 302, *d* = 1.65, ***p* < 0.001**	*U* = 406, *d* = 1.36, ***p* < 0.001**	*U* = 980.5, *d* = 0.58, *p* = 0.15
Paralarvae^a^	OMZ	10	−17.9 ± 0.4	10.1 ± 0.4	4.0 ± 0.1
	Normal	16	−18.4 ± 0.3	8.4 ± 0.3	3.9 ± 0.04
	Differences		*U* = 54, *d* = 0.63, *p* = 0.18	*U* = 22, *d* = 0.85, ***p* = 0.002**	*U* = 57.5, *d* = 0.62, *p* = 0.24
Small	OMZ	2	−18.1 ± 0.1	13.8 ± 0.8	4.18 ± 0.05
	Normal	19	−18.3 ± 0.2	8.2 ± 0.3	3.7 ± 0.1
	Differences		Not applicable^b^	Not applicable^b^	Not applicable^b^
Medium	OMZ	11	−17.9 ± 0.4	9.0 ± 0.3	3.7 ± 0.1
	Normal	14	−18.5 ± 0.1	7.4 ± 0.3	3.5 ± 0.05
	Differences		*U* = 8.5, *d* = 0.74, ***p* < 0.001**	*U* = 26, *d* = 0.71, ***p* = 0.005**	*U* = 71.5, *d* = 0.67, *p* = 0.78
Large	OMZ	20	−16.6 ± 0.1	11.9 ± 0.6	3.7 ± 0.1
	Normal	6	−18.1 ± 0.4	8.8 ± 0.2	3.5 ± 0.2
	Differences		*U* = 4.5, *d* = 0.97, ***p* < 0.001**	*U* = 26, *d* = 0.81, ***p* = 0.038**	*U* = 42, *d* = 0.62, *p* = 0.29
Atlantic specimens					
All stages	OMZ	15	−17.7 ± 0.3	9.5 ± 0.4	3.9 ± 0.1
	Normal	45	−18.4 ± 0.1	7.8 ± 0.2	3.8 ± 0.04
	Differences		*U* = 168.5, *d* = 0.80, ***p* = 0.003**	*U* = 133.5, *d* = 0.83, ***p* < 0.001**	*U* = 273, *d* = 0.60, *p* = 0.27
Paralarvae^a^	OMZ	10	−17.9 ± 0.4	10.1 ± 0.4	4.0 ± 0.1
	Normal	16	−18.4 ± 0.3	8.4 ± 0.3	3.9 ± 0.04
	Differences		*U* = 54, *d* = 0.63, *p* = 0.18	*U* = 22, *d* = 0.85, ***p* = 0.002**	*U* = 57.5, *d* = 0.62, *p* = 0.24
Small	OMZ	0	—	—	—
	Normal	16	−18.4 ± 0.2	7.8 ± 0.3	3.8 ± 0.1
	Differences		Not applicable^b^	Not applicable^b^	Not applicable^b^
Medium	OMZ	3	−17.9 ± 0.3	7.7 ± 0.5	3.5 ± 0.1
	Normal	12	−18.5 ± 0.1	7.1 ± 0.2	3.6 ± 0.1
	Differences		Not applicable^b^	Not applicable^b^	Not applicable^b^
Large	OMZ	2	−16.5 ± 0.2	8.9 ± 0.4	3.9 ± 0.3
	Normal	1	−17.3	9.0	3.8
	Differences		Not applicable^b^	Not applicable^b^	Not applicable^b^
Pacific specimens					
All	OMZ	28	−16.8 ± 0.1	12.1 ± 0.5	3.8 ± 0.1
	Normal	6	−18.7 ± 0.2	8.9 ± 0.2	3.2 ± 0.05
	Differences		*U* = 1, *d* = 1.68, ***p* < 0.001**	*U* = 36, *d* = 0.82, ***p* = 0.032**	*U* = 35, *d* = 0.82, ***p* = 0.027**
Small	OMZ	2	−18.1 ± 0.1	13.8 ± 0.8	4.18 ± 0.05
	Normal	1	−18.5	9.1	3.2
	Differences		Not applicable^b^	Not applicable^b^	Not applicable^b^
Medium	OMZ	8	−16.7 ± 0.2	11.3 ± 0.9	3.7 ± 0.2
	Normal	2	−18.62 ± 0.05	9.4 ± 0.3	3.35 ± 0.09
	Differences		Not applicable^b^	Not applicable^b^	Not applicable^b^
Large	OMZ	18	−16.7 ± 0.1	12.2 ± 0.7	3.7 ± 0.1
	Normal	3	−18.7 ± 0.5	8.5 ± 0.2	3.17 ± 0.04
	Differences		Not applicable^b^	Not applicable^b^	Not applicable^b^

Significant differences between groups are in bold.

^a^All paralarvae were sampled in the Atlantic;.

^b^comparison was not applied, if one or both columns were absent or had 3 or less values.

### Values of *δ*^15^N and trophic levels of *Vampyroteuthis infernalis*

While the overall range of *δ*^15^N was 10.2‰ (i.e. mean ± SE was 9.4 ± 0.2‰), the TLs were only from 3.0 to 4.3 (3.7 ± 0.04; Fig. 2B, Table 1). Trophic levels showed no significant geographic differences (*H*_2,104_ = 1.03, *d* = 0.20, *p* = 0.60). A significant relationship was found between habitat depth and *δ*^15^N (Spearman *r* = –0.42, *p* = 0.002), but not TL (Spearman *r* = 0.05, *p* = 0.71). This suggests that TLs do not change with depth, and this is supported by the finding of no significant depth-related TL trends within any of the three oceans.

Values of *δ*^15^N showed a significant ontogenetic increasing trend with high dispersion (Pearson *r* = 0.26, *p* = 0.009). The highest values were detected in large specimens (11.0 ± 0.5‰), and differed significantly overall (*F*_3,100_ = 6.36, *d* = 0.96, *p* < 0.001), and from relatively high values in paralarvae (9.1 ± 0.3‰; *Q* = 4.36, *d* = 0.86, *p* = 0.011) and from the lowest values in small (8.7 ± 0.4‰; *Q* = 5.14, *d* = 0.92, *p* = 0.003) and medium (8.7 ± 0.5‰; *Q* = 5.23, *d* = 0.87, *p* = 0.002) specimens. In contrast, TLs decreased significantly (Pearson *r* = –0.34, *p* < 0.001), with the highest values in paralarvae (3.9 ± 0.04), followed by small specimens (3.8 ± 0.1), large specimens (3.7 ± 0.1) and finally the lowest values in medium-sized specimens (3.6 ± 0.1). The two latter groups differed significantly from paralarvae (*F*_3,100_ = 4.83, *d* = 0.81, *p* = 0.003; *Q* = 5.03, *d* = 1.14, *p* = 0.003 and *Q* = 4.21, *d* = 0.52, *p* = 0.021, respectively).

Both *δ*^15^N values and TL showed significant ontogenetic decreases in the Atlantic (Pearson *r* = –0.42, *p* < 0.001 for *δ*^15^N, and Pearson *r* = −0.50, *p* < 0.001 for TL). Patterns for both *δ*^15^N and TL within the Atlantic fitted that for general TL, with paralarvae having the highest values and medium specimens having the lowest (Table 1). Significant differences were found only between these two groups (*H*_3,66_ = 17.29, *d* = 1.10, *p* < 0.001 and *U* = 66.5, *d* = 1.94, *p* = 0.001 for *δ*^15^N; *H*_3,66_ = 19.64, *d* = 1.21, *p* < 0.001 and *U* = 43, *d* = 2.29, *p* < 0.001 for TL). No significant relationships were found in the Pacific (Pearson *r* = −0.04, *p* = 0.80 and Pearson *r* = −0.21, *p* = 0.23, respectively). Values of *δ*^15^N in the Pacific fitted the pattern found in the Atlantic, while large specimens (as opposed to medium in the Atlantic) had the lowest TLs (Table 1). No significant differences in *δ*^15^N and TL were found among groups in the Pacific. Four available specimens from the Indian Ocean were included for general comparisons only; they fitted the established relationships of ontogenetic decrease in *δ*^15^N values in the Atlantic and Pacific Oceans (Table 1). Thus, the coefficient of variation of TL increases during ontogenesis, globally and in the Atlantic (Table 1).

In relation to OMZs, the lowest values of *δ*^15^N were found in specimens caught in ‘normal’ oxygenation conditions (8.1 ± 0.2‰), followed by OLZs (8.9 ± 0.5‰) and the highest in OMZs (11.2 ± 0.4‰), a similar pattern to *δ*^13^C (Table 2, Supplementary Table 3). Differences were found between specimens from ‘normal’ and OMZs (*H*_2,104_ = 32.10, *d* = 1.30, *p* < 0.001; *U* = 406, *d* = 1.36, *p* < 0.001), and were repeated in all ontogenetic groups, overall and from the Atlantic and the Pacific (Table 2, Supplementary Table 3): a similar pattern to that seen in *δ*^13^C. Trophic levels did not show significant differences between specimens from ‘normal’ and OMZs on a global scale and in the Atlantic, while values for the OMZ were significantly higher in the Pacific (*U* = 35, *d* = 0.82, *p* = 0.027) (Table 2, Supplementary Table 3).

### Isotopic niches of *Vampyroteuthis infernalis*

**Figure 3:**
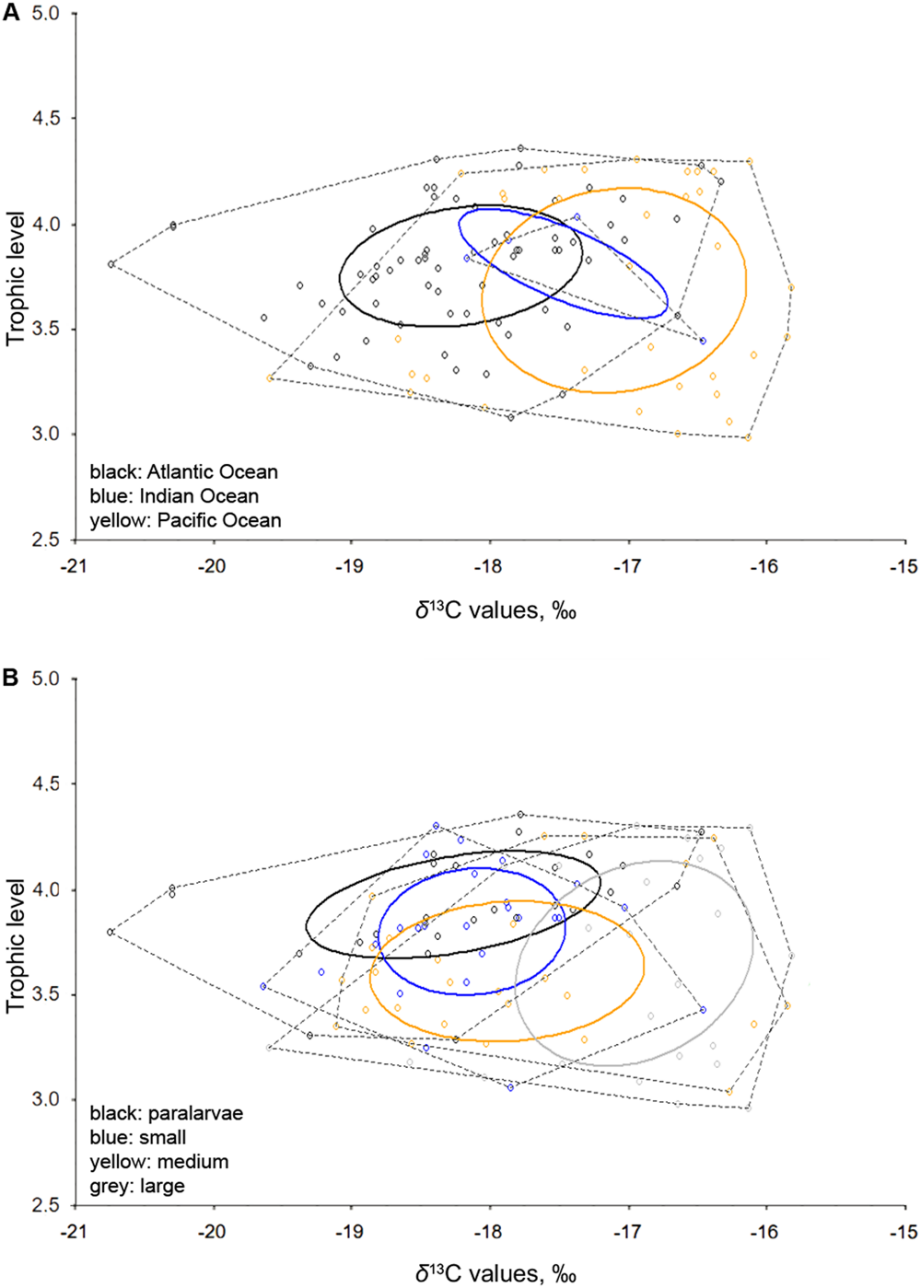
Stable isotopic niches of *Vampyroteuthis infernalis*. (**A**) Geographic approach. (**B**) Ontogenetic approach.

The narrowest niche was in the Indian Ocean, as indicated by both Layman metric of convex hull area (TA) and standard ellipse area corrected for small sample sizes (SEAc), while the widest was in the Pacific (Fig. 3A). However, the Indian Ocean (TA = 0.34, SEAc = 0.65) was represented by four specimens only, which obviously influenced (i.e. underestimated) the outcome. The niche of the Atlantic specimens (TA = 3.63, SEAc = 0.79) was significantly narrower than that of the Pacific (TA = 3.62, SEAc = 1.50) (Bayesian approximation of the standard ellipse area, SEAb: 0.74 ± 0.10 vs 1.44 ± 0.26, *p* < 0.001). Ontogenetically, large specimens had the widest niche, followed by medium specimens, and the narrowest niches were found in paralarvae and small specimens (Fig. 3B, Table 3). Niches of both large and medium specimens were significantly different from both small specimens and paralarvae (Table 3).

**Table 3:** Isotopic niche metrics for ontogenetic groups in *Vampyroteuthis infernalis*, and respective differences in niche widths.

Stage	Paralarvae	Small	Medium	Large
n	27	24	26	27
TA	2.75	2.19	2.94	3.27
SEAc	0.79	0.66	1.06	1.32
SEAb	0.63 ± 0.12	0.63 ± 0.13	1.11 ± 0.23	1.17 ± 0.24
Paralarvae	—	0.513	**0.981**	**0.990**
Small	0.487	—	**0.977**	**0.985**
Medium	**0.020**	**0.023**	—	0.573
Large	**0.010**	**0.015**	0.427	—

Significant differences between groups are in bold.

## Discussion

*Vampyroteuthis infernalis* has perplexed biologists since it was first described: the peculiar anatomy^8–11^ challenged our understanding of its phylogenetic position^12–16^, and the strong ecological association with the OMZs has initiated an interest in their habitat preferences^18–20,23–25^. The global analysis of *V. infernalis* presented here has shown that the species has a dynamic, inverted ontogenetic trajectory in *δ*^15^N values and TL, coupled with a niche broadening instead of narrowing. It occupies the same TL (3.0–4.3) over its global range and has a unique niche as an opportunistic detriti- and zooplanktivore in deep-sea ecosystems.

*Vampyroteuthis infernalis* follows the expected latitudinal pattern of *δ*^13^C values in the Pacific, decreasing latitudinally from the equator towards the poles. In the Atlantic, *δ*^13^C values were more variable and apparently unrelated to latitude. While not strictly following the expected latitudinal baseline patterns in some areas of the Atlantic^46,47^, zooplankton and top predators tend generally to follow it on an ocean-wide scale^46,48–50^. The same pattern of *δ*^13^C values, barely varying with latitude, has also been demonstrated from sampling of deep-sea sharks predominantly taken in the North Atlantic^35^. Typically, surface and bottom distributions of *δ*^13^C are known to decrease latitudinally from the equator towards the poles^48–50^. Species from epipelagic and neritic ecosystems, including cephalopods, typically follow these patterns of increased *δ*^13^C values^33–36,44^. In contrast, deepwater species do not strictly follow the known *δ*^13^C baseline patterns in the regions studied. The *δ*^13^C values for *V. infernalis* reported here are congruent with those of earlier reports^37,38^. A significant ontogenetic increase of *δ*^13^C in *V. infernalis*, coupled with lack of significant depth-related trends, suggests that there are no ontogenetic depth preferences in *V. infernalis*.

Values of *δ*^15^N for *V. infernalis* in our study followed POM baselines^51,52^, as is the case for the epipelagic and neritic marine species that have been studied to date (e.g.^34,36^). The only possible limitation of our study was the lack of information on short-term temporal isotope variations. However, the nearest possible time to our sampling period was chosen for every sample from our baseline distribution models used^51,52^ to counter this issue (see Methods). The overall broad range of values of *δ*^15^N (i.e. 10.2‰) with a high maximum (16.1‰; among cephalopod beaks, second only to the large predatory squid *Dosidicus gigas*, which is 17.7‰^45^) can be misleading to imply a consequent ontogenetic TL increase and overall high TL. We conclude that raw *δ*^15^N values, though useful for regional studies (e.g.^33,42–45^), are misrepresentative when making global scale comparisons, and it is advisable instead to use TL as an adjustment to any region-specific baseline. Also, depending on whether beaks or muscle-based tissues are used for measurements, further adjustments for the TL of cephalopods are necessary (see Methods and^33,41,53,54^). In estimating the TL of cephalopods, pelagic Antarctic Tunicata are the taxonomic group most commonly used for baseline data (e.g.^40,41,55^). This choice is considered inappropriate by some scientists^56^, as recent results suggest that tunicates are selective feeders and part of the microbial food chain^56^ and references therein. The choice of baseline coupled with the absence of beak measurement correction^33,41,53,54^ (this study) partly explains previous overestimates of TL for *V. infernalis*^40^, as well as lower TLs in predatory squids^41^, compared to the TL values for *Vampyroteuthis* presented here.

A significant ontogenetic increase in both *δ*^15^N and TL is well known for predatory squid species, e.g. *Gonatus fabricii*^44^ (Fig. 2B) and many others^33,42,43,45,57^. The ontogenetic increase in *δ*^15^N obtained here for *V. infernalis* is obviously an effect from the high variation among baseline *δ*^15^N values both between and within oceans, as TL estimations revealed a clear ontogenetic decrease even in the pooled data. Non-pooled data showed a decrease in both *δ*^15^N and TL in the Atlantic and Pacific (although only significantly for the Atlantic), and in all the locations where the minimum number of specimens was five (i.e. Nova Scotia, Gulf of Mexico, central east Atlantic, Pacific side of Australia, California and Peru). The only exceptions were specimens from Ecuadorian waters. These results reveal a well-supported general ontogenetic decrease of *δ*^15^N and TL in *V. infernalis*.

Applying our muscle tissue corrections (see Methods) to *δ*^15^N values for *V. infernalis* obtained by other authors^37–39^ (Seibel *et al*., unpublished data) gives TLs that fit with our observations of higher *δ*^15^N values and TLs in smaller specimens. An exception was a series of unusually low values found in specimens from the Bay of Biscay^37^. However, the beaks used in that study were obtained from sperm whales^37^ that may have obtained their prey from regions with a higher *δ*^15^N baseline than that of the Bay of Biscay, thus rendering the baseline data questionable.

The TL of *V. infernalis* remained the same with depth, thus the observed significant *δ*^15^N decrease with depth requires further explanation: it could have resulted from material limitations and comparison of all the oceans together. Due to biogeochemical degradation, it is known that POM increases its *δ*^15^N values with depth, and that POM-eating plankton and micronekton show a similar increase following that of POM, especially among those that do not undergo daily vertical migrations^39,58–60^. In contrast, planktophagous and predatory species lack such an increase with depth^39,59,60^. The lack of an increase in *δ*^15^N values with depth in *V. infernalis* could therefore be explained by this species consuming food other than solely detritus, as observed in stomach contents analysis^18^, a deduction also supported by other data (discussed below).

*Vampyroteuthis infernalis* has a higher TL than that of other studied pelagic POM-feeders but it is lower than those of high-level predators (cf.^34,37–39,41,59–61^). In terms of classical trophic ecology, *V. infernalis* has been observed eating either marine aggregates including POM or large prey (mesoplanktonic crustaceans and gelatinous taxa), and the remains of both have been found in its stomach contents^8,18^ (Seibel, pers. comm.). Apart from those taxa, fish scales and bones, fragments of squid flesh, a few protozoan groups, and diatoms were recorded among the stomach contents of *V. infernalis*^8,18^. Thus, stomach contents analyses give a contradictory picture regarding species diet (all known observations summarized in Supplementary Table 2). Moreover, all the studies rely on a limited number of specimens and all are from closely located areas of the North Pacific^8,18^ (Seibel, pers. comm.). Applying other methods (i.e. stable-isotope analysis) clarifies the contradictions shown by stomach contents analysis (reasons beyond ontogenetic changes in TL and food spectrum are explained in the next paragraph below). Data united from stomach contents and stable isotope analyses demonstrate that the trophic ecology of *V. infernalis* is unique among cephalopods, which in contrast are opportunistic predators or zooplankton feeders^6,29^. Judging by its high TL for a pelagic POM-eater, it is clear that *V. infernalis* benefits from the crustaceans and other small-sized animals that are associated with marine snow. The consumption of organisms associated with POM by *V. infernalis* has been suggested before for specimens from southern California, Monterey Bay and Mexico^18^ and our SIA data show that this unique trophic link applies to the diet of the species globally. This mode of feeding coupled with occasional carrion consumption would explain the high TLs observed here. Additional proof that *V. infernalis* is not a ‘purely’ detritivorous, planktivorous or carnivorous species is as follows. Scavenging crustaceans, amphipods and isopods, show much higher TLs than carnivorous and detritivorous invertebrates from the same ecosystems (cf.^62–64^). Thus, with its seemingly contradictory stomach contents^8,18^ and a TL higher than other studied pelagic POM-feeders but lower than high-level predators (cf.^34,37–39,41,59–61^), *V. infernalis* has a mixed food spectrum and unique feeding mode.

Higher values of *δ*^15^N and TL in paralarvae and small specimens than in medium and large specimens indicate that ontogenetic changes in *V. infernalis* foraging are unique among the cephalopods studied to date. The explanation may lie in changing the mode of locomotion during ontogenesis, which is known for *V. infernalis*^20,28^. Paralarvae have the highest *δ*^15^N and TL values since they rely more on plankton-feeding. Capture of other planktonic animals is facilitated by the relatively fast and agile movements supplied by jet propulsion (expelling water forcibly through the funnel, characteristic of all coleoid cephalopods^28^). Small specimens continue to use jet propulsion during a period of ‘gait transition’, when they subsequently switch to fin propulsion^28^. Then, they would rely more on POM, which is easier to obtain considering the more passive movements of their new gait. High *δ*^15^N and TL values in small specimens indicate that they prefer, and can still obtain, plankton. Larger specimens, moving only by fin propulsion, become more flexible and opportunistic in their feeding habits and their niche becomes significantly wider (Fig. 3B, Table 3). The highest coefficients of variation of TL in large specimens is indeed indicative of the proposed flexibility in their diet, which is also reflected in their stomach contents^8,18^ (Seibel, pers. comm.). They presumably begin to eat larger planktonic forms, detritus and carrion at this size category. *Vampyroteuthis infernalis* occupies the same TL worldwide, while in purely predatory species it varies in different areas of the range^36^. However, in view of the paucity of published global studies, it is not known if this is generally characteristic of deepwater species, of deepwater pelagic POM-feeders, or unique to *V. infernalis*.

The presence of an OMZ has been noted in tropical areas of the Atlantic, Pacific and Indian Oceans, usually on their eastern sides^65,66^. This means that much of the range of *V. infernalis* does not involve an OMZ. Values of both *δ*^13^C and *δ*^15^N are higher in individuals analyzed from an OMZ than outside one, overall, in the Atlantic and in the Pacific. That is found for all ontogenetic groups, proving that it is not a sample-based bias or a mixing of ontogenetic differences and OMZ-related differences. Nevertheless, their respective TL values were the same overall and in the Atlantic. There are no SIA studies with which we might compare our results. The baselines of *δ*^15^N were higher in OMZs^51,52^, and to a lesser degree also for *δ*^13^C^49,50^. An increase in geographic area and upper depth limits of OMZs have been reported recently as one possible consequence of climate change^66–68^. Among the extinct relatives of *V*. *infernalis*, there were large pelagic and possibly benthic species, carnivorous and with a lifestyle possibly resembling that of Recent squids^69–71^. Only *V. infernalis* has survived until recent times, and the OMZ seems to have played a significant role in the success of this relict from the past, perhaps as a refugium. There are fewer predators present in the OMZs^18,66^, so evolving a feeding mode that enables access to an abundant food source (POM with associated organisms) in an otherwise harsh environment with low oxygen levels is important. In the OMZs *V. infernalis* can escape competition with other cephalopods and avoid predators. This unique trophic ecology distinguishes *V. infernalis* from the other coleoid cephalopods and, coupled with its ability to live in the OMZs, this relict species may be at a competitive advantage in the current conditions of ongoing climate change.

## Methods

### Sampling and measurements

A total of 144 *V. infernalis* beaks were analyzed from 104 specimens collected in the Atlantic, Pacific and Indian Oceans (Fig. 1) between 1905 and 2017 (66 specimens collected between 2007 and 2017). The estimated mantle lengths (ML) ranged from 6 to 198 mm (Table 1, Supplementary Table 1). Specimens obtained from the stomach contents of predators (*n* = 20) were randomly allocated within a 1000 km uncertainty radius around a predator’s suggested capture location. *Vampyroteuthis infernalis* (with a life span of possibly more than 5 years^72^) grows continuously throughout its life and its size at maturity is highly variable^20,73,74^. Therefore, the specimens were categorized into four arbitrary ontogenetic groups: paralarvae, ML < 27 mm; small, ML 27–65 mm; medium, ML 66–104 mm; and large, ML > 105 mm. Shortly after hatching, *V. infernalis* has two pairs of swimming fins^20,28^. At about 25 mm ML, one pair (the hatchling fins) are resorbed, leaving a single pair of fins^20,28^. Thus, division of large paralarvae from small specimens was also based on the presence of four fins or corresponded to the size at which four fins were still present^20,28^ if the voucher animal was not available for examination. Division of small, medium and large specimens roughly corresponded to juvenile, maturing and mature size distribution, as close as it was possible according with the known sizes of ontogenetic stages^20,74^.

Beak dimensions, lower hood length (LHL) and upper hood length (UHL), were measured following Clarke^73,75^. A total of 20 beaks were obtained from the stomach contents of predators, and the ML of many fixed or frozen specimens was not measured (*n* = 28) or unreliably measured (*n* = 37). Only the upper beak was available from 33 specimens (Supplementary Table 1). Existing published equations to estimate the ML of *V. infernalis* from beak size are either based on regional studies or on specific ontogenetic groups with small samples sizes^20,73,75,76^. Therefore, new equations were derived to estimate ML from LHL (1) and UHL (2), and these are used in the present study (all *p* < 0.001):

1 $${\rm{ML}}=6.131\ast {{\rm{LHL}}}^{1.2908},{r}^{2}=0.80,p=0.00001,n=27({\rm{ML}}\,11\mbox{--}125\,{\rm{mm}});$$

2 $${\rm{ML}}=7.3646\ast {\rm{UHL}}-9.0676,{r}^{2}=0.77,p=0.00001,n=50\,({\rm{ML}}\,11\mbox{--}125\,{\rm{mm}}).$$

In addition, a photographic record of the form of the lower and upper beaks at all ontogenetic stages of *V. infernalis* collected across the world are provided (Supplementary Fig. 1).

Estimations from LHL were preferred to UHL where both beaks were available because lower beaks are preferred for SIA^33^ and remain more often in the stomach contents of predators^77^. When only upper beaks were available (*n* = 33), the stable isotope values of the missing lower beaks were estimated by calibration equations (all *p* < 0.001) based on analysis of *δ*^13^C and *δ*^15^N for specimens with both beaks intact:

3 $${\delta }^{13}{{\rm{C}}}_{{\rm{lower}}{\rm{beak}}}=0.8169\ast {\delta }^{13}{{\rm{C}}}_{{\rm{upper}}{\rm{beak}}}-3.449,{r}^{2}=0.91,p=0.00001,n=24({\rm{ML}}\,6\mbox{--}170\,{\rm{mm}});$$

4 $${\delta }^{15}{{\rm{N}}}_{{\rm{lower}}{\rm{beak}}}=1.4066\ast {\delta }^{15}{{{\rm{N}}}_{{\rm{lower}}{\rm{beak}}}}^{0.8408},{r}^{2}=0.84,p=0.00001,n=24\,({\rm{ML}}\,6\mbox{--}170\,{\rm{mm}}).$$

Therefore, the results reported here refer only to actual or estimated values for lower beaks. Raw values for lower and upper beaks are shown in Supplementary Table 1. Most of the beaks were from fixed specimens, but neither ethanol nor formalin fixation affects significantly *δ*^13^C or *δ*^15^N measurements^54^, and no corrections were performed. Values of *δ*^15^N in cephalopod beaks, in contrast to *δ*^13^C values, are typically lower (around 4.8‰ on average) than values obtained from muscle tissue^33,41,53,54^. Therefore, ‘muscle’ values of *δ*^15^N available in the literature were reduced by 4.8‰ to enable comparison with the data reported here. However, when estimating TL, this value was restored to our ‘beak’ values of *δ*^15^N, following^41,44^.

### Stable isotope analysis

All beaks were dried at 60 °C for 24–48 hours and ground into a fine powder. Powder sub-samples were weighed (to the nearest 0.3 mg) with a micro-balance and sterile-packed in tin containers. Stable isotope values were determined by a Flash EA 1112 Series elemental analyser coupled online via a Finnigan ConFlo II interface to a Delta VS mass spectrometer (Thermo Scientific) and expressed as: *δ*^13^C and *δ*^15^N = [(R_sample_/R_standard_) − 1] * 1000, where R = ^13^C/^12^C and ^15^N/^14^N, respectively. The carbon and nitrogen isotope ratios were expressed in delta (*δ*) notation relative to Vienna-PeeDee Belemnite limestone (V-PDB) for *δ*^13^C and atmospheric nitrogen (AIR) for *δ*^15^N. Replicate measurements of internal laboratory standards (acetanilide STD: Thermo Scientific PN 338 36700) in every batch (*n* = 14) indicated precision <0.2‰ for both *δ*^13^C and *δ*^15^N values. Mean mass C:N ratio of all samples was 3.19 ± 0.02.

### Data analyses

Values of *δ*^13^C and *δ*^15^N, and TL, were examined and compared within and among geographic areas (Pacific, Atlantic and Indian Oceans), ontogenetic stages (paralarvae, small, medium and large specimens) and in relation to O_2_ concentration in water masses (OMZ, OLZ and ‘normal’). Normality of data distribution was checked with Kolmogorov–Smirnov and Shapiro–Wilk tests^78^. Mantle length was square-root transformed to fit normality^78^. Data were analyzed using an ANOVA or a Kruskal–Wallis *H* test, with further pairwise multiple comparisons using a Tukey’s HSD test or Mann–Whitney *U* test^78^. Effect size (Cohen’s *d*) was calculated by standard procedures where applicable^79^. Coefficients of variation were estimated for TL in ontogenetic groups as a measure of within-group variability^78^. A regression analysis was used to estimate the equations fitting the data, and any relationships between the variables were assessed using a Pearson correlation or Spearman’s rank correlation^78^. All tests were performed using a significance value of *α* = 0.05.

There is no universal definition of OMZ in relation to O_2_ concentration^65,66^. It is here defined as areas where the oxygen concentration is <20 μmol kg^−1^ in the Pacific and Indian Oceans or <45 μmol kg^−1^ in the Atlantic Ocean, following^66^. Specimens caught within these conditions were described as living in the OMZ. Specimens caught in adjacent areas, with concentrations of O_2_ higher than that defined for the OMZ but lower than ‘normal’ oceanic oxygen concentration (i.e.<60 μmol kg^−1^ in the Pacific and Indian Oceans or <90 μmol kg^−1^ in the Atlantic Ocean), are referred to as from the OLZ^66^. Other parts of the oceans, with oxygen concentration > 60 μmol kg^−1^ in the Pacific and Indian Oceans or >90 μmol kg^−1^ in the Atlantic Ocean, are referred to as ‘normal’ oxygenation conditions. The areas with different oxygen concentration were taken from^65^ and^66^. In addition, depth-related changes in *δ*^13^C and *δ*^15^N values, and TLs were explored where depth of capture was available (*n* = 55). Depths of capture were estimated as mean values between net opening and net closing, if both were available (*n* = 17), otherwise only the single recorded value was used.

Trophic level was estimated by using a classical Eq. (5)^80^:

5 $${{\rm{TL}}}_{V.infernalis}=[({\delta }^{15}{{\rm{N}}}_{V.infernalis}\,\mbox{--}\,{\delta }^{15}{{\rm{N}}}_{{\rm{POM}}{\rm{baseline}}})/{\rm{TEF}}]+{{\rm{TL}}}_{{\rm{POM}}{\rm{baseline}}}.$$

The classical trophic enrichment factor (TEF) value of 3.4 was used for the world ocean^30^ because estimation of additional parameters worldwide (such as the TL-related saturating isotope limit, the rate at which stable isotope value approaches a forementioned limit per TL step, and slope and intercept values from appropriate meta-analyses for every region) is required to use a scaled approach to TEF^81,82^. It is known that pelagic animals in the upper and core regions of the OMZs rely mostly on epipelagic POM, while those in deeper regions rely mostly on deepwater POM^83^. Accordingly, to obtain a baseline, for each sampling locality we derived epipelagic POM baseline values^51^ and bottom POM baseline values^52^, and determined mean values between epipelagic and bottom POM. Both models cover our sampling time^51,52^, thus the nearest possible time to our sampling period was chosen for every sample.

Stable isotopic niche widths of different groups were analyzed using the recent metrics based in a Bayesian framework (Stable Isotope Bayesian Ellipses in R: SIBER^84^), which allows robust statistical comparisons. The standard ellipse area corrected for small sample sizes (SEAc, an ellipse that contains 40% of the data regardless of sample size) and the Layman metric of convex hull area (TA) were estimated using the software package SIBER under R 3.5.0 (R Core Team 2018; see^84,85^). The Bayesian approximation of the standard ellipse area (SEAb) was adopted to compare niche width between groups (i.e. p, the proportion of ellipses in one group that were lower than in another group; see^84^ for more details). Sample size was larger than the smallest recommended for SIBER^86^ in all ontogenetic groups and overall in the Atlantic and Pacific Oceans. Trophic levels were used instead of *δ*^15^N values (Y axis) to calculate niche space. According to classical definition, a niche is a multivariate space with coordinates showing bionomic and scenopoetic ecological information (i.e. habitat usage and trophic level^85,87^). Thus, *δ*^13^C and *δ*^15^N are usually taken as axes^84,85,87^. Since TL is a way to improve the ecological meaning of the bionomic data^41,81^ which are highly variable due to *δ*^15^N baseline variations worldwide^51,52^, our approach to niche analyses is fully justified in the context of a global study. It has already been applied to niche analyses of Arctic cephalopods elswhere^88^.

Statistical analysis, calculations and plots were performed with Statistica 10.0 (Statsoft), PAST 3.15^89^ and MS Excel 2010. Values are presented as mean ± SE, unless otherwise stated.

### Ethical approval

No ethical approval was required. Beaks were obtained only from preserved specimens already deposited in museum, university and research collections. No live animals were caught for this project. Responsible curators of collections in respective museums, or material owners from respective universities and research institutes were participating in the project, thus all necessary permissions were obtained.

## Supplementary information

Supplementary Information

## Data Availability

All relevant data are included in the paper and/or in the supplementary information (Supplementary Table 1).
