# Supplementary material for: The first global deep-sea stable isotope assessment reveals the unique trophic ecology of Vampire Squid Vampyroteuthis infernalis (Cephalopoda)
Source: Sci Rep. 2019 Dec 13;9:19099. doi: 10.1038/s41598-019-55719-1 (PMC6910912; doi:10.1038/s41598-019-55719-1)
Supplement: Supplementary file 1 — Supplementary Information [file 41598_2019_55719_MOESM1_ESM.pdf]

Supplementary materials for the article

The first global deep-sea stable isotope assessment reveals the unique trophic ecology of Vampire Squid *Vampyroteuthis infernalis* (Cephalopoda)

Alexey V. Golikov*, Filipe R. Ceia, Rushan M. Sabirov, Jonathan D. Ablett, Ian G. Gleadall, Gudmundur Gudmundsson, Hendrik J. Hoving, Heather Judkins, Jónbjörn Pálsson, Amanda L. Reid, Rigoberto Rosas-Luis, Elizabeth K. Shea, Richard Schwarz & José C. Xavier

*Corresponding author: golikov_ksu@mail.ru

Supplementary Table 1. Biometric measurements (ML, mantle length; LHL and UHL, lower and upper hood length), ontogenetic stage, stable isotope values of lower beaks ($\delta^{13}C$ and $\delta^{15}N$) and estimated trophic level (TL) and collection area information for all specimens of *Vampyroteuthis infernalis* used in this study

Number	Ocean	Depth, m[1]	Oxygenation conditions[2]	Specimen condition	ML, mm (accepted)[3]	Stage	LHL, mm	UHL, mm	$\delta^{13}C$, ‰[4]	$\delta^{15}N$, ‰[4]	TL
PS0722-1	Atlantic	1400.0	Normal	Fixed[5]	19	Paralarva	–	6.53	–18.9	7.3	3.7
PS0722-2	Atlantic	1400.0	Normal	Fixed[5]	24	Paralarva	–	6.09	–17.8	7.7	3.9
PS0722-3	Atlantic	1400.0	Normal	Fixed[5]	23	Paralarva	–	7.16	–18.4	7.1	3.7
PS0765-3	Atlantic	700.0	Normal	Fixed[5]	20	Paralarva	–	5.72	–20.3	8.1	4.0
PS1466-2	Atlantic	1355.0	Normal	Fixed[5]	17	Paralarva	–	4.02	–18.8	7.4	3.8
PS1466-3	Atlantic	1355.0	Normal	Fixed[5]	15	Paralarva	–	3.68	–18.4	8.5	4.1
PS2132-3	Atlantic	1439.0	Normal	Fixed[5]	14	Paralarva	–	4.23	–18.2	8.5	4.1
PS2132-4	Atlantic	1439.0	Normal	Fixed[5]	21	Paralarva	–	6.68	–19.4	7.1	3.7
PS2598-3	Atlantic	1395.0	Normal	Fixed[5]	22	Paralarva	–	4.83	–18.5	7.7	3.9
PS2598-4	Atlantic	1395.0	Normal	Fixed[5]	23	Paralarva	3.54	5.18	–18.4	7.4	3.8
PS2425-2	Atlantic	1327.0	Normal	Fixed[5]	11	Paralarva	2.63	3.95	–18.4	8.7	4.2
PS0765-2	Atlantic	700.0	Normal	Fixed[5]	40	Small	–	6.87	–18.8	7.2	3.7
PS1448-1	Atlantic	1437.0	Normal	Fixed[5]	49	Small	–	6.17	–19.3	5.9	3.3
PS1448-2	Atlantic	1437.0	Normal	Fixed[5]	27	Small	–	5.01	–18.5	8.7	4.2
PS1466-1	Atlantic	1355.0	Normal	Fixed[5]	42	Small	–	6.1	–18.1	8.4	4.1
PS2132-1	Atlantic	1439.0	Normal	Fixed[5]	42	Small	–	6.85	–18.5	7.5	3.8

PS2132-2	Atlantic	1439.0	Normal	Fixed[5]	33	Small	–	7.13	–18.7	7.5	3.8
PS1600-1	Atlantic	1418.0	Normal	Fixed[5]	36	Small	–	6.82	–18.6	6.4	3.5
PS1600-2	Atlantic	1418.0	Normal	Fixed[5]	40	Small	–	9.82	–19.6	6.5	3.5
PS1600-3	Atlantic	1418.0	Normal	Fixed[5]	39	Small	–	10.82	–18.2	6.6	3.5
PS1334-1	Atlantic	1271.0	Normal	Fixed[5]	31	Small	–	7.4	–18.5	7.5	3.8
PS1291-1	Atlantic	1448.0	Normal	Fixed[5]	31	Small	–	4.87	–18.4	9.2	4.3
PS1358-1	Atlantic	1143.0	Normal	Fixed[5]	51	Small	–	8.26	–19.2	6.8	3.6
PS0765-1	Atlantic	700.0	Normal	Fixed[5]	77	Medium	–	9.35	–18.9	7.2	3.7
PS1902-1	Atlantic	724.0	Normal	Fixed[5]	93	Medium	–	12.53	–18.9	6.2	3.4
PS1334-2	Atlantic	1271.0	Normal	Fixed[5]	66	Medium	–	6.82	–18.8	8.0	4.0
PS1291-2	Atlantic	1448.0	Normal	Fixed[5]	77	Medium	–	8.62	–18.7	7.3	3.8
PS2383-1	Atlantic	1319.0	Normal	Fixed[5]	66	Medium	–	13.31	–19.1	5.9	3.3
PS2406-1	Atlantic	1417.0	Normal	Fixed[5]	69	Medium	–	11.92	–18.3	5.9	3.4
PS2598-1	Atlantic	1395.0	Normal	Fixed[5]	82	Medium	–	13.31	–18.8	6.8	3.6
PS2598-2	Atlantic	1395.0	Normal	Fixed[5]	79	Medium	–	9.85	–19.1	6.6	3.6
PS2425-1	Atlantic	1327.0	Normal	Fixed[5]	84	Medium	–	13.85	–17.9	6.5	3.5
TEM2007-768-21	Atlantic	792.0	Normal	Fixed[6]	18	Paralarva	3.45	–	–17.5	9.4	3.9
TEM2007-768-58	Atlantic	250.0	Normal	Fixed[6]	17	Paralarva	3.48	–	–16.7	9.7	4.0
TEM2007-768-64	Atlantic	1000.0	Normal	Fixed[6]	22	Paralarva	2.61	3.87	–17.0	10.0	4.1
TEL2010-900-40	Atlantic	750.0	Normal	Fixed[6]	25	Paralarva	2.9	4.03	–17.4	9.3	3.9
TEM2007-768-8	Atlantic	1195.0	Normal	Fixed[6]	53	Small	5.3	–	–17.9	9.4	3.9
NED2009-035-32	Atlantic	1750.0	Normal	Fixed[6]	40	Small	4.72	–	–17.0	9.4	3.9
NED2009-035-46	Atlantic	1250.0	Normal	Fixed[6]	38	Small	4.66	–	–17.8	9.2	3.9
TEM2007-768-13	Atlantic	1694.0	Normal	Fixed[6]	88	Medium	7.28	–	–17.9	7.8	3.5
TEL2010-900-17	Atlantic	250.0	Normal	Fixed[6]	84	Medium	5.85	–	–17.6	8.2	3.6
TEL2010-900-55	Atlantic	1337.0	Normal	Fixed[6]	105	Large	7.44	–	–17.3	9.0	3.8
A11-2016-355(1)	Atlantic	775.0	Normal	Fixed[7]	63	Small	5.9	7.2	–17.5	9.2	3.9
675	Atlantic	800.0	OMZ	Frozen[8]	9	Paralarva	1.31	2.87	–18.0	9.8	3.9
D10379-19	Atlantic	–	Normal	Fixed[9]	6	Paralarva	0.92	1.36	–20.3	10.1	4.0
20150263	Atlantic	–	OMZ	Fixed[9]	24	Paralarva	2.9	4.4	–16.5	11.1	4.3
12183-10	Atlantic	–	OMZ	Fixed[9]	25	Paralarva	2.98	3.95	–17.3	10.7	4.2
D6622-16	Atlantic	–	OMZ	Fixed[9]	13	Paralarva	1.18	2.88	–20.7	9.4	3.8
D7089-08	Atlantic	–	OMZ	Fixed[9]	15	Paralarva	1.98	3.81	–18.3	7.7	3.3
D9541-30	Atlantic	–	OMZ	Fixed[9]	7	Paralarva	1.1	2.02	–17.8	11.6	4.3

20150264	Atlantic	–	OMZ	Fixed[9]	9	Paralarva	1.29	1.92	–17.5	10.5	4.1
20150261	Atlantic	–	OMZ	Fixed[9]	16	Paralarva	2.1	3.1	–17.8	11.8	4.3
D7803-2	Atlantic	–	OMZ	Fixed[9]	12	Paralarva	1.68	3	–17.1	10.1	4.0
D7824-34	Atlantic	–	OMZ	Fixed[9]	26	Paralarva	–	4.81	–18.5	8.6	3.8
D8553-2	Atlantic	–	OLZ	Fixed[9]	26	Paralarva	3.06	4.17	–18.1	10.2	3.9
12	Atlantic	490.0	OLZ	Frozen[8]	33	Small	3.71	6.11	–17.9	7.4	3.0
676	Atlantic	800.0	OLZ	Frozen[8]	37	Small	4	7.68	–17.5	9.7	3.9
D8558-1B	Atlantic	–	OLZ	Fixed[9]	27	Small	3.13	3.31	–18.1	9.6	3.7
677	Atlantic	800.0	OLZ	Frozen[8]	104	Medium	9.01	12.9	–17.8	8.6	3.8
D10378-24	Atlantic	–	Normal	Fixed[9]	87	Medium	7.81	10.04	–18.3	8.1	3.6
D7803-11	Atlantic	–	OMZ	Fixed[9]	89	Medium	7.97	10.79	–17.4	8.4	3.5
D7824-19	Atlantic	–	OMZ	Fixed[9]	70	Medium	6.59	8.39	–18.4	8.0	3.7
D7824-2	Atlantic	–	OMZ	Fixed[9]	74	Medium	6.86	8.92	–18.0	6.6	3.3
D7089-5	Atlantic	–	OMZ	Fixed[9]	133	Large	10.87	14.93	–16.6	8.6	3.5
D7803-31	Atlantic	–	OMZ	Fixed[9]	128	Large	10.56	13.89	–16.3	9.3	4.2
D85262-6	Atlantic	–	OLZ	Fixed[9]	120	Large	10.04	14.32	–17.5	7.8	3.2
D1739	Indian	–	Normal	Fixed[9]	62	Small	5.97	8.22	–16.5	11.2	3.4
D5332	Indian	–	Normal	Fixed[9]	59	Small	5.79	6.76	–18.2	10.0	3.8
SA-1	Indian	–	Normal	Whale[10]	134	Large	10.93	–	–17.4	9.2	4.0
SA-2	Indian	–	Normal	Whale[10]	145	Large	11.62	–	–17.9	8.8	3.9
AM C.451587	Pacific	660.0	Normal	Fixed[11]	61	Small	5.96	–	–18.5	9.1	3.2
AM C.154223	Pacific	640.0	Normal	Fixed[11]	73	Medium	6.83	–	–18.6	9.1	3.3
AM C.450195	Pacific	800.0	Normal	Fixed[11]	69	Medium	6.5	–	–18.7	9.7	3.4
AM C.451584	Pacific	640.0	Normal	Fixed[11]	126	Large	10.43	–	–18.0	8.6	3.1
AM C.451586	Pacific	485.0	Normal	Fixed[11]	152	Large	12.04	–	–18.6	8.8	3.2
J-1	Pacific	–	Normal	Bird[12]	124	Large	10.3	–	–19.6	8.1	3.2
P-8	Pacific	–	OMZ	Whale[10]	101	Medium	8.77	–	–17.3	13.2	3.3
P-1	Pacific	–	OMZ	Whale[10]	138	Large	11.14	–	–17.0	14.9	3.8
P-2	Pacific	–	OMZ	Whale[10]	134	Large	10.9	–	–16.4	13.1	3.3
P-3	Pacific	–	OMZ	Whale[10]	134	Large	10.93	–	–16.5	16.1	4.1
P-4	Pacific	–	OMZ	Whale[10]	132	Large	10.78	–	–16.4	15.2	3.9
P-5	Pacific	–	OMZ	Whale[10]	138	Large	11.15	–	–16.9	15.8	4.0
P-6	Pacific	–	OMZ	Whale[10]	124	Large	10.29	–	–16.6	12.9	3.2
P-7	Pacific	–	OMZ	Whale[10]	139	Large	11.25	–	–16.4	12.8	3.2

P-9	Pacific	–	OMZ	Whale[10]	155	Large	12.21	–	–16.6	8.2	3.0
P-10	Pacific	–	OMZ	Whale[10]	198	Large	14.79	–	–15.8	10.6	3.7
R-1	Pacific	–	OMZ	Shark[13]	96	Medium	8.4	–	–16.1	8.4	3.4
R-6	Pacific	–	OMZ	Shark[13]	80	Medium	–	12.03	–15.9	8.8	3.4
R-7	Pacific	–	OMZ	Shark[13]	85	Medium	–	12.73	–16.3	7.4	3.0
R-2	Pacific	–	OMZ	Shark[13]	110	Large	9.37	–	–16.8	8.6	3.4
R-3	Pacific	–	OMZ	Shark[13]	170	Large	13.12	–	–17.5	11.0	4.1
R-4	Pacific	–	OMZ	Shark[13]	130	Large	10.67	–	–16.9	7.5	3.1
R-5	Pacific	–	OMZ	Shark[13]	113	Large	9.56	–	–16.1	7.1	3.0
549	Pacific	652.8	OMZ	Frozen[8]	27	Small	3.16	5.2	–17.9	14.6	4.1
563	Pacific	–	OMZ	Frozen[8]	49	Small	5.02	8.43	–18.2	12.9	4.2
576	Pacific	–	OMZ	Frozen[8]	73	Medium	6.82	8.84	–17.3	13.5	4.3
531	Pacific	–	OMZ	Frozen[8]	66	Medium	6.27	13.21	–17.6	13.0	4.2
572	Pacific	–	OMZ	Frozen[8]	92	Medium	8.18	12.75	–16.6	13.1	4.1
553	Pacific	581.3	OMZ	Frozen[8]	79	Medium	7.23	12.55	–16.4	13.5	4.2
569	Pacific	–	OMZ	Frozen[8]	110	Large	9.39	13.73	–17.9	12.5	4.1
561	Pacific	–	OMZ	Frozen[8]	111	Large	9.43	15.95	–16.5	13.0	4.2
540	Pacific	–	OMZ	Frozen[8]	112	Large	9.51	15.6	–16.9	13.2	4.3
533	Pacific	–	OMZ	Frozen[8]	116	Large	9.75	16.6	–16.1	13.6	4.3
550	Pacific	745.7	OMZ	Fixed[8]	124	Large	10.29	15.65	–16.6	13.5	4.2

[1] depth of capture was estimated as mean value between net opening and net closing, if both were available (see Methods);

[2] oxygen conditions were classified as oxygen minimum zone (OMZ), oxygen limited zone (OLZ) and 'normal' (Normal) (see Methods);

[3] accepted ML: the accepted ML was estimated from LHL or UHL for beaks with no measured ML or no reliably measured ML (see Methods);

[4] $\delta^{13}C$ and $\delta^{15}N$: for samples where only upper beaks were available, stable isotope values of lower beaks were estimated by highly significant equations relating upper and lower beaks derived from specimens where both beaks were analyzed (see Methods);

[5] fixed specimens vouchered in Department of Biological Sciences, University of South Florida St. Petersburg;

[6] fixed specimens vouchered in Delaware Museum of Natural History (Wilmington);

[7] fixed specimen vouchered in Collections and Systematics Department, Icelandic Institute of Natural History (Gardabaer). Northernmost record of species distribution;

[8] fixed or frozen specimens vouchered in GEOMAR, Helmholtz Centre for Ocean Research Kiel;

[9] fixed specimens vouchered in Department of Life Sciences, Natural History Museum (London);

[10] beaks from predator stomach contents: stomach of sperm whales, *Physeter macrocephalus* (Clarke 1980);

[11] fixed specimens vouchered in the Australian Museum (Sydney);

[12] beak from predator stomach contents: stomach of Antipodean wandering albatrosses, *Diomedea antipodensis antipodensis* (Xavier et al. 2014);

[13] beaks from predator stomach contents: stomach of blue sharks, *Prionace glauca* (Loor-Andrade et al. 2017).

Cited literature:

Clarke, M. R. Cephalopoda in the diet of sperm whales of the southern hemisphere and their bearing on sperm whale biology. *Discovery Rep.* **37**, 1–324 (1980).

Loor-Andrade, P., Pincay-Espinoza, J. & Rosas-Luis, R. Diet of the blue shark *Prionace glauca* in the Ecuadorian Pacific Ocean during the years 2013 to 2015. *J. Appl. Ichthyol.* **33**, 558–562 (2017).

Xavier, J. C., Walker, K., Elliott, G., Cherel, Y. & Thompson, T. Cephalopod fauna of South Pacific waters: new information from breeding New Zealand wandering albatrosses. *Mar. Ecol. Prog. Ser.* **513**, 131–142 (2014).

Supplementary Table 2. Known stomach contents and observations of feeding of *Vampyroteuthis infernalis*.

Phylum	Class	Species	Organism composition	Observation mode	Area	Reference
–	–	–	Detritus	Stomach contents analysis	Monterey Bay	Hoving & Robison 2012
				ROV observations	Monterey Bay	Hoving & Robison 2012
Ochrophyta	Bacillariophyceae	Various	Whole and fragments	Stomach contents analysis	San Pedro Basin off southern California	Young 1977
					Monterey Bay	Hoving & Robison 2012
Retaria	Radiolaria	Various	Whole and fragments	Stomach contents analysis	San Pedro Basin off southern California	Young 1977
					Monterey Bay	Hoving & Robison 2012
Ciliophora	Not stated	Various	Whole and fragments	Stomach contents analysis	Monterey Bay	Hoving & Robison 2012
Cnidaria	Not stated	Hydrozoan or/and scyphozoan medusae	Fragments	Stomach contents analysis	Monterey Bay	Hoving & Robison 2012
Ctenophora	Not stated	Various	Fragments	Stomach contents analysis	Monterey Bay	Hoving & Robison 2012
Artrhropoda	Not stated	Crustacean larvae	Whole and fragments	Stomach contents analysis	San Pedro Basin off southern California	Young 1977
		Small non-larval crustaceans	Fragments	Stomach contents analysis	San Pedro Basin off southern California	Young 1977
					Monterey Bay	Hoving & Robison 2012
		Large non-larval crustaceans	Fragments	Stomach contents analysis	Monterey Bay	Hoving & Robison 2012
	Ostracoda	Various	Whole and fragments	Stomach contents analysis	Monterey Bay	Hoving & Robison 2012

	Maxillopoda	Copepods	Whole, fragments and fecal pellets	Stomach contents analysis	San Pedro Basin off southern California	Young 1977
					Monterey Bay	Hoving & Robison 2012
	Malacostraca	Amphopods	Whole and fragments	Stomach contents analysis	Monterey Bay	Hoving & Robison 2012
Mollusca	Cephalopoda	*Histioteuthis heteropsis*	Whole squid, attacked and ingested[1]	Direct observation of feeding in aquaria	San Clemente Basin off southern California	Siebel, pers. comm.
		Gonatopsis borealis	Fragments	Stomach contents analysis	Monterey Bay	Hoving & Robison 2012
Chordata	Larvacea	Different species	Faecal pellets, fragments and discarded mucus filtration houses	Stomach contents analysis	Monterey Bay	Hoving & Robison 2012
		Bathocordaeus sp.	Discarded mucus filtration houses	Stomach contents analysis	Monterey Bay	Hoving & Robison 2012
	Thaliacea	*Doliolum* sp.	Fragments and discarded mucus filtration houses	Stomach contents analysis	Monterey Bay	Hoving & Robison 2012
	Osteichthyes	Unidentified	Scales and bones	Stomach contents analysis	Monterey Bay	Hoving & Robison 2012

[1] clearly not a typical feeding behavior, as stated by author of the observation.

Cited literature:

Young, J. Z. Brain, behaviour and evolution of cephalopods. *Symp. Zool. Soc. Lond.* **38**, 377–434 (1977).

Hoving, H. J. T. & Robison, B. H. Vampire squid: detritivores in the oxygen minimum zone. *Proc. R. Soc. Ser. B – Bio.* **279**, 4559–4567 (2012).

Supplementary Table 3. Mantle length (ML), values of δ^{13}C and δ^{15}N and estimated trophic level (TL) in *Vampyroteuthis infernalis* from different oxygenation conditions: oxygen minimum zones (OMZ), oxygen limited zones (OLZ) and 'normal' (Normal) (see Methods for O_2 concentrations). Values are minimum – maximum (mean ± standard error)

Stage	All specimens, OMZ					All specimens, OLZ[1]				
	n	ML, mm	δ^{13}C, ‰	δ^{15}N, ‰	TL	n	ML, mm	δ^{13}C, ‰	δ^{15}N, ‰	TL
All	43	7–198 (88.1 ± 7.8)	–20.7– –15.8 (–17.1 ± 0.1)	6.6–16.1 (11.2 ± 0.4)	3.0–4.3 (3.8 ± 0.1)	6	26–120 (57.9 ± 17.4)	–18.1– –17.5 (–17.8 ± 0.1)	7.4–10.2 (8.9 ± 0.5)	3.0–3.9 (3.6 ± 0.2)
Paralarvae	10	7–26 (15.5 ± 2.3)	–20.7– –16.5 (–17.9 ± 0.4)	7.7–11.8 (10.1 ± 0.4)	3.3–4.3 (4.0 ± 0.1)	1	26	–18.1	10.2	3.9
Small	2	27–49 (38.1 ± 16.1)	–18.2– –17.9 (–18.1 ± 0.1)	12.9–14.6 (13.8 ± 0.8)	4.1–4.2 (4.18 ± 0.05)	3	27–37 (32.2 ± 2.9)	–18.1– –17.5 (–17.8 ± 0.2)	7.4–9.7 (8.9 ± 0.7)	3.0–3.9 (3.5 ± 0.2)
Medium	11	66–124 (55.3 ± 10.4)	–18.4– –15.9 (–17.9 ± 0.4)	6.6–11.1 (9.0 ± 0.3)	3.0–4.3 (3.7 ± 0.1)	1	104	–17.8	8.6	3.8
Large	20	110–198 (132.6 ± 4.9)	–17.9– –15.8 (–16.6 ± 0.1)	7.1–16.1 (11.9 ± 0.6)	3.0–4.3 (3.7 ± 0.1)	1	120	–17.5	7.8	3.2
Stage	All specimens, Normal					Atlantic specimens, OMZ				
	n	ML, mm	δ^{13}C, ‰	δ^{15}N, ‰	TL	n	ML, mm	δ^{13}C, ‰	δ^{15}N, ‰	TL
All	55	6–152 (54.8 ± 14.9)	–20.3– –16.5 (–18.4 ± 0.1)	5.9–11.2 (8.1 ± 0.2)	3.1–4.3 (3.7 ± 0.04)	15	7–133 (43.3 ± 11.4)	–20.7– –16.3 (–17.7 ± 0.3)	6.6–11.8 (9.5 ± 0.4)	3.3–4.3 (3.9 ± 0.1)
Paralarvae	16	6–25 (18.5 ± 1.3)	–20.3– –16.7 (–18.4 ± 0.3)	7.1–10.1 (8.4 ± 0.3)	3.7–4.2 (3.9 ± 0.04)	10	7–26 (15.5 ± 2.3)	–20.7– –16.5 (–17.9 ± 0.4)	7.7–11.8 (10.1 ± 0.4)	3.3–4.3 (4.0 ± 0.1)
Small	19	27–63 (44.0 ± 2.6)	–19.6– –16.5 (–18.3 ± 0.2)	5.9–11.2 (8.2 ± 0.3)	3.2–4.3 (3.7 ± 0.1)	0	–	–	–	–
Medium	14	66–93 (78.1 ± 2.3)	–19.1– –17.6 (–18.5 ± 0.1)	5.9–9.7 (7.4 ± 0.3)	3.3–4.0 (3.5 ± 0.05)	3	70–89 (77.6 ± 6.0)	–18.4– –17.4 (–17.9 ± 0.3)	6.6–8.4 (7.7 ± 0.5)	3.3–3.7 (3.5 ± 0.1)
Large	6	105–152 (131.2 ± 6.9)	–19.6– –17.3 (–18.1 ± 0.4)	8.1–9.2 (8.8 ± 0.2)	3.1–4.0 (3.5 ± 0.2)	2	128–133 (130.9 ± 2.4)	–16.6– –16.3 (–16.5 ± 0.2)	8.6–9.3 (8.9 ± 0.4)	3.5–4.2 (3.9 ± 0.3)
Stage	Atlantic specimens, OLZ[1]					Atlantic specimens, Normal				
	n	ML, mm	δ^{13}C, ‰	δ^{15}N, ‰	TL	n	ML, mm	δ^{13}C, ‰	δ^{15}N, ‰	TL

All	6	26–120 (57.9 ± 17.4)	–18.1– –17.5 (–17.8 ± 0.1)	7.4–10.2 (8.9 ± 0.5)	3.0–3.9 (3.6 ± 0.2)	45	6–105 (44.6 ± 4.0)	–20.3– –16.7 (–18.4 ± 0.1)	5.9–10.1 (7.8 ± 0.2)	3.3–4.3 (3.8 ± 0.04)
Paralarvae	1	26	–18.1	10.2	3.9	16	6–25 (18.5 ± 1.3)	–20.3– –16.7 (–18.4 ± 0.3)	7.1–10.1 (8.4 ± 0.3)	3.7–4.2 (3.9 ± 0.04)
Small	3	27–37 (32.2 ± 2.9)	–18.1– –17.5 (–17.8 ± 0.2)	7.4–9.7 (8.9 ± 0.7)	3.0–3.9 (3.5 ± 0.2)	16	27–63 (40.9 ± 2.3)	–19.6– –17.0 (–18.4 ± 0.2)	5.9–9.4 (7.8 ± 0.3)	3.3–4.3 (3.8 ± 0.1)
Medium	1	104	–17.8	8.6	3.8	12	66–93 (79.3 ± 2.5)	–19.1– –17.6 (–18.5 ± 0.1)	5.9–8.2 (7.1 ± 0.2)	3.3–4.0 (3.6 ± 0.1)
Large	1	120	–17.5	7.8	3.2	1	105	–17.3	9.0	3.8
Stage	Pacific specimens, OMZ					Pacific specimens, Normal				
	n	ML, mm	δ^{13}C, ‰	δ^{15}N, ‰	TL	n	ML, mm	δ^{13}C, ‰	δ^{15}N, ‰	TL
All	28	27–198 (112.0 ± 6.8)	–18.2– –15.8 (–16.8 ± 0.1)	7.1–16.1 (12.1 ± 0.5)	3.0–4.3 (3.8 ± 0.1)	6	61–152 (101.0 ± 15.2)	–19.6– –18.0 (–18.7 ± 0.2)	8.1–9.7 (8.9 ± 0.2)	3.1–3.4 (3.2 ± 0.05)
Paralarvae	0	–	–	–	–	0	–	–	–	–
Small	2	27–49 (38.1 ± 11.1)	–18.2– –17.9 (–18.1 ± 0.1)	12.9–14.6 (13.8 ± 0.8)	4.1–4.2 (4.18 ± 0.05)	1	61	–18.5	9.1	3.2
Medium	8	66–101 (83.8 ± 4.2)	–17.6– –15.9 (–16.7 ± 0.2)	7.4–13.5 (11.3 ± 0.9)	3.0–4.3 (3.7 ± 0.2)	2	69–73 (70.9 ± 2.3)	–18.7– –18.6 (–18.62 ± 0.05)	9.1–9.7 (9.4 ± 0.3)	3.3–3.4 (3.35 ± 0.09)
Large	18	110–198 (132.8 ± 5.4)	–17.9– –15.8 (–16.7 ± 0.1)	7.1–16.1 (12.2 ± 0.7)	3.0–4.3 (3.7 ± 0.1)	3	124–152 (134.2 ± 9.0)	–19.6– –18.0 (–18.7 ± 0.5)	8.1–8.8 (8.5 ± 0.2)	3.1–3.2 (3.17 ± 0.04)

[1] all specimens from OLZ were sampled in the Atlantic.

Supplementary Fig. 1. Images of lower and upper beaks of *Vampyroteuthis infernalis* obtained from all ontogenetic stages. Lower beaks (side, oblique and facing views): A, paralarva (northeast Atlantic Ocean, Guinea, ML 13 mm); B, small (equatorial area of the Atlantic Ocean, ML 27 mm); C, medium (southwest Pacific Ocean, southeast Australia, ML 73 mm); D, large (central east Pacific Ocean, Peru, ML 139 mm). Upper beaks (side, oblique and top views): E, small (northeast Atlantic Ocean, Guinea, ML 27 mm); F, medium (central east Pacific Ocean, Ecuador), ML 85 mm); G, large (northeast Atlantic Ocean, Cape-Verde, ML 133 mm). Scale bars: 1 mm. All photos are original. Beak views terminology follows Lu & Ickeringill 2002.

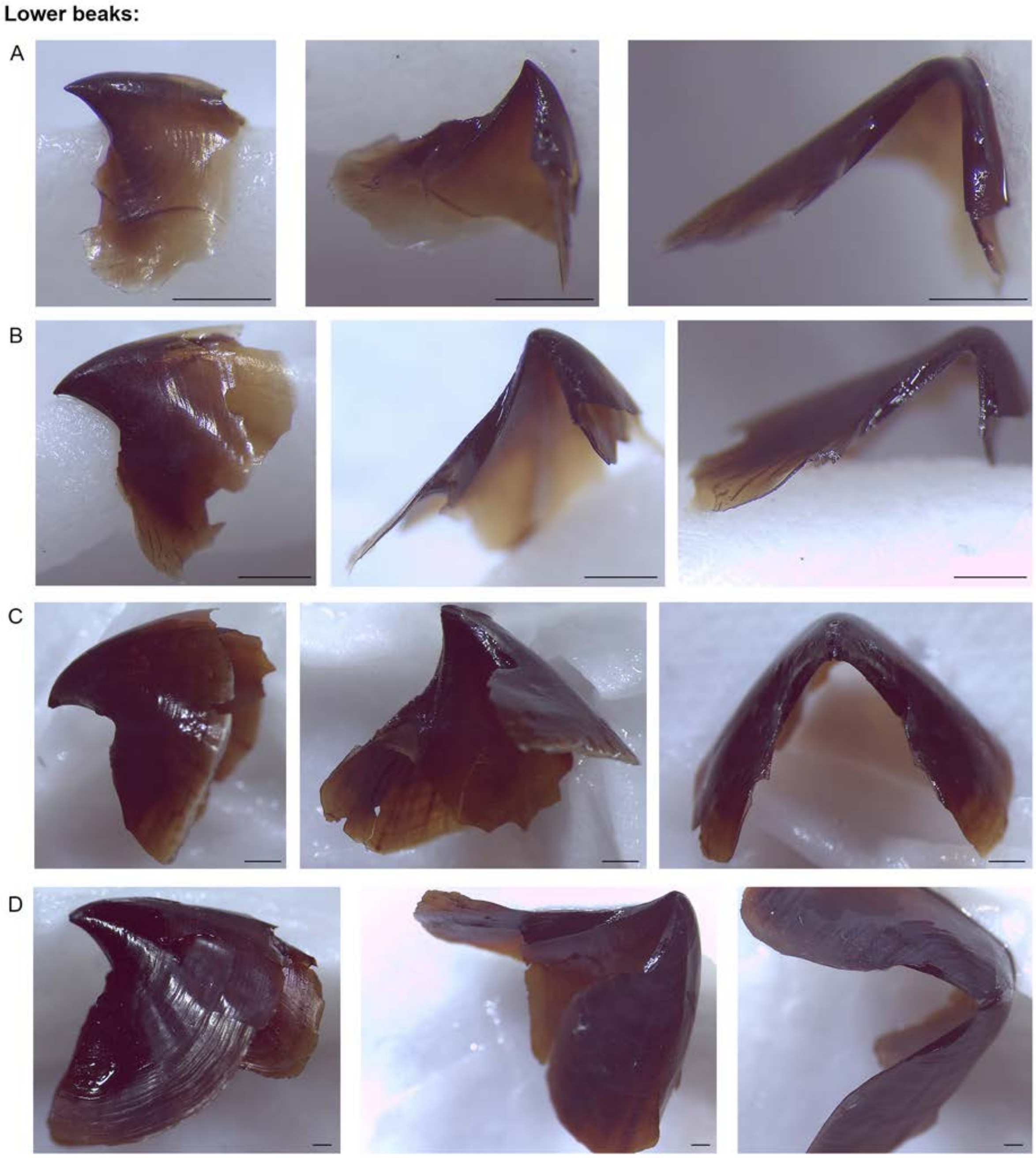

Upper beaks:

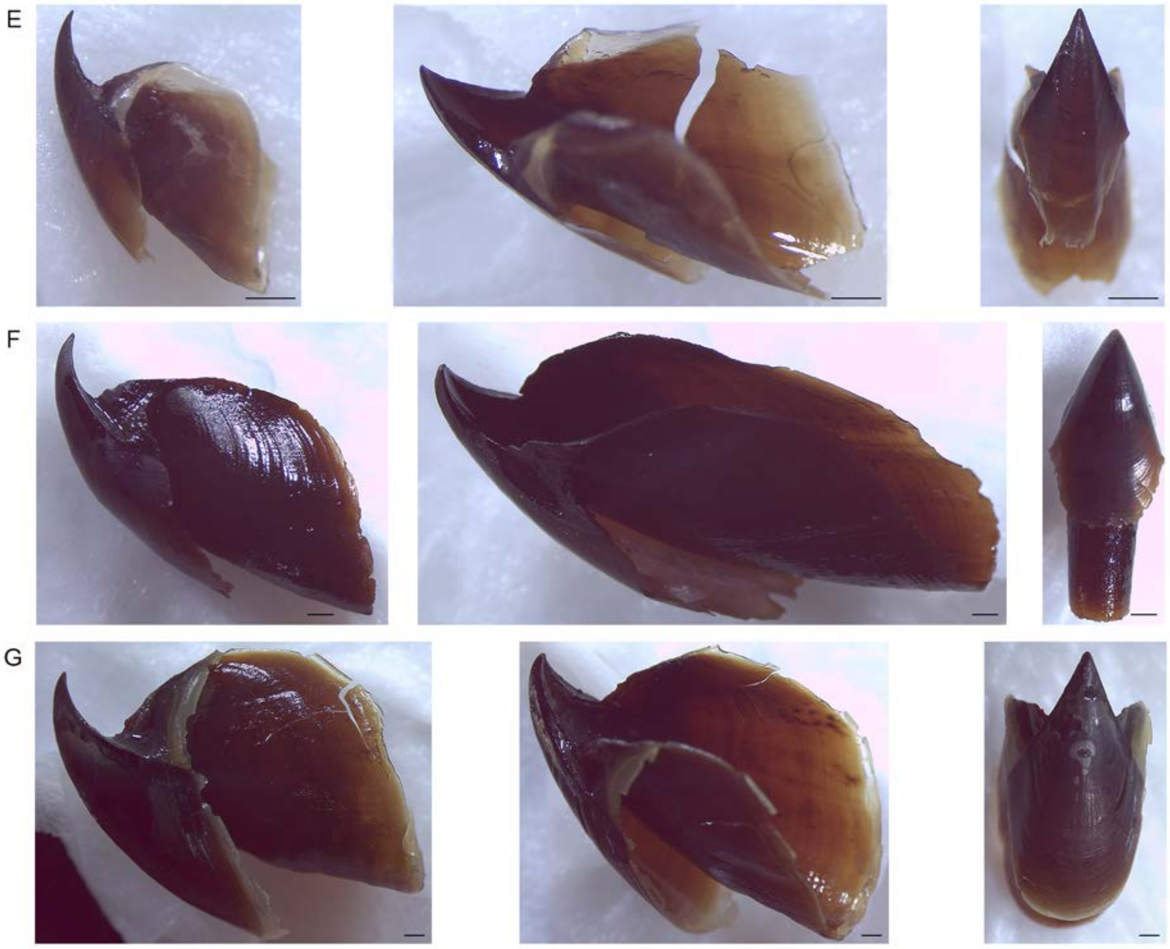

Cited literature:

Lu, C. C. & Ickeringill, R. Cephalopod beak identification and biomass estimation techniques: tools for dietary studies of southern Australian finfishes. *Mus. Vic. Sci. Rep.* **6**, 1–65 (2002)
